# A buccal mucosa carcinoma treated with high dose rate brachytherapy

**DOI:** 10.1120/jacmp.v6i1.2064

**Published:** 2005-03-17

**Authors:** Joann I. Prisciandaro, Robert L. Foote, Michael G. Herman, Sally J. Lee, Wayne N. LaJoie, Andrew B. Van Blarcom, Peter D. Yeakel

**Affiliations:** ^1^ Division of Radiation Oncology Mayo Clinic 200 First Street SW Rochester Minnesota 55905; ^2^ Prosthodontics, Mayo Clinic 200 First Street SW Rochester Minnesota 55905; ^3^ Present address: Division of Radiation Oncology University of Michigan Medical Center 1500 E. Medical Center Dr. Ann Arbor Michigan 48109 U.S.A.; ^4^ Present address: Radiation Oncology Abbott Northwestern Hospital 800 E. 28th St. Minneapolis Minnesota 55402 U.S.A.; ^5^ Present address: Varian Brachytherapy Charlottesville VA 22903 U.S.A.; ^6^ Present address: 5000 West 95th St., Suite 290 Prairie Village KS 66207 U.S.A.

**Keywords:** brachytherapy, buccal mucosa carcinoma

## Abstract

This manuscript presents a case of early stage squamous cell carcinoma of the left buccal mucosa treated with intensity‐modulated radiation therapy (IMRT) followed by a high‐dose rate (HDR) brachytherapy boost. With limited literature available on HDR mold (stent) radiotherapy for oral cancer, a discussion on the issues encountered during treatment planning and delivery may prove to be insightful for facilities faced with a similar challenge.

PACS numbers: 87.53.Jw, 87.53.Tf, 87.59.Ci, 87.59.Fm

## I. INTRODUCTION

Squamous cell carcinoma of the buccal mucosa is relatively rare in the United States and Western Europe and comprises approximately 10% of all carcinomas of the oral cavity.^(1)^ For early stage lesions, T1 and T2, treatment options include surgery or external beam radiation therapy followed by a localized boost with an electron beam, interstitial implant, or intra‐oral cone.^(2)^ Studies have shown radiation therapy to provide comparable results to surgery^(3)^ ^,^ ^(4)^ with improved cosmesis and function. Although brachytherapy has proven to be a viable treatment option, the literature has focused on the use of interstitial brachytherapy.^(^ ^4^ ^–^ ^7^ ^)^ Limited data is available on high‐dose rate (HDR) mold/stent radiation therapy for the treatment of oral cancer.^(8)^

This work presents a case of early stage squamous cell carcinoma of the left buccal mucosa treated with intensity‐modulated radiation therapy (IMRT) followed by an HDR brachytherapy boost. The initial treatment was delivered with IMRT to spare the contralateral parotid and oral cavity mucosa in order to prevent xerostomia and minimize acute mucositis.

## II. METHODS AND MATERIALS

For this patient, the intention was to immediately follow IMRT (total dose of 60 Gy to the entire buccal mucosa and 54 Gy to the ipsilateral neck nodes in 30 fractions over 6 weeks) with an HDR boost to the left buccal mucosa. External beam radiotherapy was administered prior to HDR to minimize the tumor's thickness.

Prior to beginning the HDR course of treatment, it was necessary to create an applicator through which the radioactive source could be transported. A custom acrylic radiation stent (density^(9)^ ~1.19g/cm ^3^) was constructed prior to treatment in prosthodontics to conform to the patient's lower jaw and attach to his teeth for fixation. The left central section of the stent was extended laterally to minimize dose to the tongue and mandible. Four blind‐ended catheters spaced approximately 5 mm apart were laid horizontally over this thickened portion of the stent to provide uniform coverage to the tumor area. A spacing of 5 mm was selected to control the dose distribution delivered by this nonplanar stent and to ensure ample coverage in case of a compromised catheter. The catheters were then covered by an additional 5 mm of acrylic to reduce the dose to the mucosal surface. The stent was then modified to ensure a comfortable and reproducible fit. Several adjustments were necessary before an adequate fit was achieved. This step was essential to produce a fixed geometry applicator for which a pretreatment plan could be generated.

Once the applicator was constructed, the treatment was simulated by acquiring images of the patient with the stent in place. A strand of dummy markers was inserted into each catheter. Each strand consisted of a series of radio‐opaque markers separated by a distance of 1 cm. The markers are used to mimic potential source positions in each catheter and assist with catheter reconstruction. The treatment was simulated with both computed tomography (CT) and via fluoroscopic images from the Nucletron® BV integrated brachytherapy unit. Although a CT‐based treatment plan was desired, the artifacts generated by the patient's fillings and the dummy markers rendered this technique challenging, at best. Due to time constraints, only the fluoroscopic images were utilized for treatment planning. A second CT scan of the stent alone in a container of water was acquired to compare the dose distribution between the two simulation modalities after the completion of treatment.

**Figure 1:**
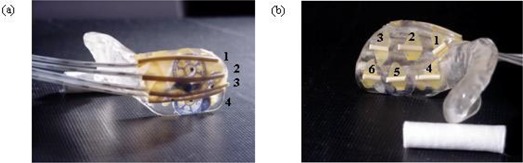
Lateral and inner surface view of the custom‐made stent. (a) Wires were placed over the position of each catheter to define the lateral surface of the stent. Four bee bees were also positioned on the lateral surface to denote the anterior, posterior, superior, and inferior extent of the lesion. (b) In addition, six shorter wires were placed on the inner surface of the stent.

Treatment planning was performed with the Plato brachytherapy planning system version 14.2.5 (Nucletron® BV, Veenendaal, the Netherlands). A total dose of 10 Gy was to be delivered over the course of 5 days, in 2‐Gy daily fractions, at a depth of 5 mm from the surface of the stent. However, it was quickly realized that the surface of the stent could not easily be discerned with two‐dimensional imaging. As a result, a second set of fluoroscopic images of the stent alone was obtained. Before these images were acquired, radio‐opaque wires were positioned on the lateral surface of the stent directly over the position of each catheter and on the inner surface of the stent to determine dose to tongue and mandible (Fig. 1). In addition, four bee bees were affixed to the lateral surface of the stent to denote the anterior, posterior, superior, and inferior extent of the lesion.

In the planning software, the four lateral wires were digitized as four additional catheters. Dose points were placed 5 mm to the left of each dwell position in these “catheters.” The wires along the inner surface of the applicator were digitized as marker points in order to determine the maximum dose to the tongue and mandible. The four bee bees were also digitized as markers to monitor the dose along the circumference of the lesion. Lastly, applicator points were placed 5 mm to the left of the tip, midsection, and anterior end of the central two “catheters” (lateral wires). The dose was prescribed to the applicator points (5 mm from the surface of the stent), and to achieve the desired dose cloud around the catheters, the plan was optimized on dose points. The plan was then fine‐tuned by manually adjusting the weights of the individual dwells to achieve an optimal dose distribution.

The last step in the treatment process was the actual delivery. The catheter lengths were verified on the first day of treatment with a check ruler, and to ensure the integrity of the catheters prior to each treatment, the inner core of each of the catheters was wiped. The detection of fluid on the wipe was an indication of a compromised catheter. To verify the position of the stent, semi‐orthogonal images were acquired before each treatment and compared with in vivo integrated brachytherapy unit images obtained during simulation.

Following treatment delivery, the axial CT images of the stent in water were imported into the planning system. The stent was contoured and the catheters were reconstructed with the assistance of anterior and lateral scout films. To compare the dose distributions of the fluoro‐versus the CT‐based plan, the axes for both plans were centered on the anterior bee bee and aligned such that the superior bee bee was intercepted in the *y‐z* plane and the posterior bee bee in the *x‐z* plane. The average 3D deviation for the active sources between the two plans was 0.9 mm (maximum of 1.5 mm).

## III. RESULTS

The design of the HDR stent was one of the key steps toward developing a successful treatment plan. A custom stent was designed to fit comfortably and reproducibly within the patient's mouth. Several modifications were necessary before this was accomplished. One of the main issues that arose during the construction of the stent was designing a common exit route for all four catheters such that the patient did not accidentally clamp down and compromise any of the catheters. This was an issue with the two most inferior catheters. In the early stent designs, the inferior catheters were tucked under the stent, along the patient's gum line. Thus, when the patient attempted to place the stent, it did not fit securely within his mouth. To correct for this, the inferior two catheters were repositioned so that they exited the stent with a steep superior curvature, allowing them to exit laterally rather than along the patient's gum line. However, during the first day of treatment, the inferior‐most catheter proved to be compromised. As a result, it was necessary to modify the treatment plan to prevent the source from residing in this catheter. This required adjusting the dwell weights in the remaining catheters to compensate for the compromised catheter. Even with this restriction, the resulting treatment plan proved to be satisfactory (Table 1).

**Table 1:** Relative dose of the marker, applicator, and dose points based on the final, optimized treatment plan. The dose is relative to the prescription dose (100%=2Gy). The marker points labeled “Wire n,” where n=1−6, represent the dose to the center of each of the shorter wires placed along the inner surface of the stent (see Fig. 1(b). The dose points listed are a sample of 60 that were placed 5 mm lateral to the surface of the applicator.

Marker points	Dose (%)	Applicator points	Dose (%)	Dose points	Dose (%)
wire 1	121.85	tip catheter 2	106.07	1	105.88
wire 2	142.23	mid catheter 2	111.57	5	111.43
wire 3	187.87	ant catheter 2	103.91	10	107.57
wire 4	49.83	tip catheter 3	81.94	15	106.56
wire 5	68.61	mid catheter 3	101.47	20	88.88
wire 6	65.47	ant catheter 3	95.05	25	69.93
post bee bee	333.81			30	68.12
inf bee bee	96.82			35	111.56
sup bee bee	238.03			40	107.32
ant bee bee	150.70			45	101.36
				50	101.47
				55	94.62
				60	85.98

**Figure 2:**
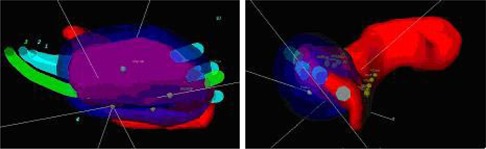
(a) Lateral and (b) posterior view of the reconstructed stent and 100% isodose cloud for the CT‐based plan

Following treatment delivery, a second treatment plan was generated from the CT scan of the stent in water. Table 2 shows a comparison of the dose points using the two simulation modalities. The dose to the wires in the CT plan was determined by entering the coordinates from the fluoroscopic plan since the wires were absent during the second CT scan. Based on these results, the dose to the lesion was found to be hotter (up to ~16%), and the dose to the tongue and mandible was found to be cooler (up to ~12.5%) than suggested by the initial fluoro‐based plan. Figure 2 depicts the dose distribution achieved by the CT plan.

**Table 2:** Comparison of dose points between the fluoro‐ and CT‐based treatment plan

Dose point	Fluoro plan dose (cGy)	CT plan dose (cGy)	ΔDose (cGy)	% Diff
post bee bee	668.98	754.36	85.38	12.76
inf bee bee	193.43	199.60	6.17	3.19
sup bee bee	474.47	549.35	74.88	15.78
ant bee bee	301.54	313.94	12.40	4.11
W1Ant	243.97	233.00	−10.97	−4.50
W1Post	243.47	235.06	−8.41	−3.45
W2Ant	273.66	263.03	−10.63	−3.88
W2Post	294.52	287.82	−6.70	−2.27
W3Ant	435.10	428.20	−6.90	−1.59
W3Post	317.61	277.98	−39.63	−12.48
W4Ant	89.36	89.17	−0.19	−0.21
W4Post	109.81	109.88	0.07	0.06
W5Ant	128.37	128.70	0.33	0.26
W5Post	146.02	145.77	−0.25	−0.17
W6Ant	145.80	143.61	−2.19	−1.50
W6Post	116.34	111.73	−4.61	−3.96

## IV. DISCUSSION

Small squamous cell carcinomas of the oral cavity are ideally suited for localized radiation therapy boost techniques, particularly in patients too ill for surgery or when surgical resection would result in unacceptable functional and/or cosmetic deficits. Cancers of the anterior tongue, floor of mouth, and buccal mucosa are especially amenable to localized boost techniques such as intra‐oral electron beam or orthovoltage cone therapy, low‐dose rate interstitial brachytherapy, or HDR interstitial brachytherapy.

Our patient had a 1.5‐cm grade 1 clinical stage I (T1, N0, M0) squamous cell carcinoma involving the left posterior and inferior aspect of the buccal mucosa. On palpation, it measured less than 5 mm in thickness and did not directly involve the gingiva, buccal space, or masseter muscle. Two independent surgical consultations suggested that surgical resection would have included wide local excision of the buccal mucosa including the buccal space and outer cheek skin, total parotidectomy, select neck dissection, and defect reconstruction using a radial fore‐arm free flap.^(10)^ The patient, who is a 47‐year‐old tile layer and drag racing mechanic and enthusiast, was concerned about trauma to and function of his forearm after free flap harvest. Therefore, after thorough evaluation and consultation in two multidisciplinary head and neck cancer clinics, he elected to proceed with primary radiation therapy. Intensity‐modulated radiation therapy was utilized to treat the clinically uninvolved ipsilateral regional lymph nodes to 54 Gy in 30 fractions of 1.8 Gy per fraction while simultaneously treating the entire buccal mucosa on the left with margin to 60 Gy in 30 fractions of 2.0 Gy per fraction. Intensity‐modulated radiation therapy was utilized to spare the contralateral parotid and submandibular glands and the majority of the intra‐oral mucosa. The patient tolerated this portion of the treatment well with the expected grade 2 dermatitis, grade 3 mucositis, and altered taste.

High‐dose rate stent brachytherapy was chosen for the localized boost technique because the lesion was located posteriorly and inferiorly in the oral cavity. The use of orthovoltage radiotherapy was briefly discussed but would require extraction of healthy teeth from the left mandible to allow docking of the cone over the lesion with adequate margins; thus this modality was dismissed as an option for this patient. A customized intra‐oral stent would also be less invasive and more comfortable for the patient when compared to interstitial implantation.

The HDR stent brachytherapy boost was administered as soon as the external beam radiation therapy was completed without interruption. The primary tumor had completely resolved during the external beam portion of the treatment, thus optimizing the HDR stent brachytherapy. An impression of the primary tumor was made on the outer surface of the stent which aided in treatment planning. The patient comfortably tolerated the 5 fractions of 2.0 Gy per fraction HDR brachytherapy for a minimal total dose of 70 Gy in 35 fractions over 7 weeks despite the localized mucositis from the external beam radiation therapy. Posttreatment biopsies of the buccal mucosa at 6 weeks and 4.5 months have been free of cancer. The mucositis resolved within 6 weeks of treatment completion. The patient's taste has returned to normal, and there is no xerostomia. The patient remains free of disease 10.5 months following treatment.

## V. CONCLUSION

High‐dose rate stent brachytherapy offers a great deal of promise for treating oral cancers. With the limited literature available on HDR stent brachytherapy, the issues encountered during our treatment planning and delivery process proved to be a good learning experience for our clinic and may be useful for facilities faced with similar challenges.
